# A Water-Soluble Sodium Pectate Complex with Copper as an Electrochemical Catalyst for Carbon Dioxide Reduction

**DOI:** 10.3390/molecules26185524

**Published:** 2021-09-11

**Authors:** Kirill V. Kholin, Mikhail N. Khrizanforov, Vasily M. Babaev, Guliya R. Nizameeva, Salima T. Minzanova, Marsil K. Kadirov, Yulia H. Budnikova

**Affiliations:** 1 Arbuzov Institute of Organic and Physical Chemistry, FRC Kazan Scientific Center, Russian Academy of Sciences, 420088 Kazan, Russia; khrizanforov@gmail.com (M.N.K.); babaev@iopc.ru (V.M.B.); minzanova@iopc.ru (S.T.M.); kamaka59@gmail.com (M.K.K.); yulia@iopc.ru (Y.H.B.); 2 Department of Nanotechnology in Electronics, Kazan National Research Technical University Named after A.N. Tupolev-KAI, 420111 Kazan, Russia; 3 Department of Physics, Kazan National Research Technological University, 420015 Kazan, Russia; guliya.riv@gmail.com

**Keywords:** pectate complex, copper, carbon dioxide reduction, electrocatalysis, methane

## Abstract

A selective noble-metal-free molecular catalyst has emerged as a fruitful approach in the quest for designing efficient and stable catalytic materials for CO_2_ reduction. In this work, we report that a sodium pectate complex of copper (PG-NaCu) proved to be highly active in the electrocatalytic conversion of CO_2_ to CH_4_ in water. Stability and selectivity of conversion of CO_2_ to CH_4_ as a product at a glassy carbon electrode were discovered. The copper complex PG-NaCu was synthesized and characterized by physicochemical methods. The electrochemical CO_2_ reduction reaction (CO_2_RR) proceeds at −1.5 V vs. Ag/AgCl at ~10 mA/cm^2^ current densities in the presence of the catalyst. The current density decreases by less than 20% within 12 h of electrolysis (the main decrease occurs in the first 3 h of electrolysis in the presence of CO_2_). This copper pectate complex (PG-NaCu) combines the advantages of heterogeneous and homogeneous catalysts, the stability of heterogeneous solid materials and the performance (high activity and selectivity) of molecular catalysts.

## 1. Introduction

The selectivity and the yields of products of the electrochemical CO_2_ reduction reaction (CO_2_RR) at room temperature, as shown by many studies, strongly depend on the material of the working electrode, on which the target reaction takes place, and the solvent used [1,2]. The main disadvantages of these heterogeneous electrochemical processes are the need to apply a relatively high potential (~−1.90 V vs. the standard hydrogen electrode (SHE)) and a low current density response. However, in an aqueous medium, the equilibrium potential of the CO_2_RR is much more positive. For example, the standard electrode potential for the reaction:

CO_2_ + H_2_O + 2e^−^ → HCOO^−^ + OH^−^ (1)

is −0.43 V vs. SHE at pH 7.0 [3].

**Figure 1:**
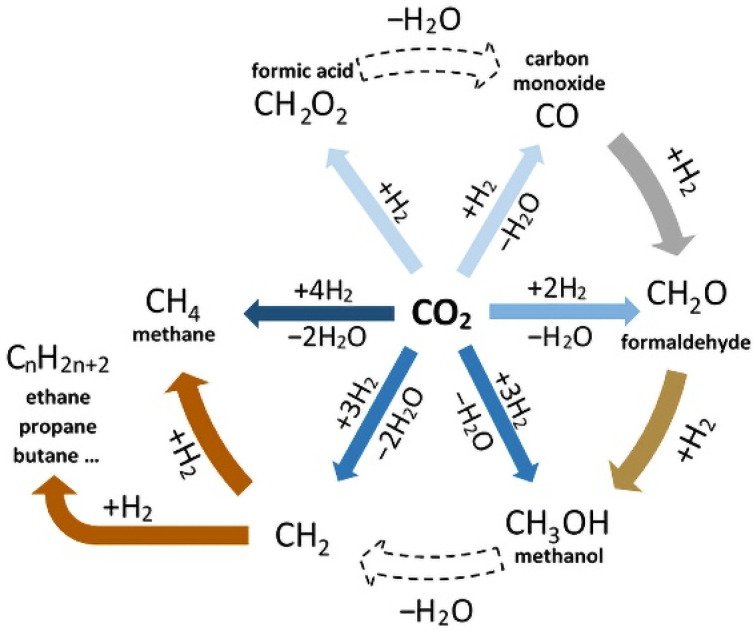
Main possible products of catalytic electrochemical reduction of CO_2_ in an aquatic environment.

However, CO_2_ reduction in water can be difficult (overpotential is more than 1 V in many cases) and the actual electrode potentials are overwhelmingly much more negative than the equilibrium potential [4]. The reason for this phenomenon is that the reaction proceeds through the formation of an intermediate—the radical anion CO_2_^−^ at an extremely negative potential. These problems can be eliminated by using efficient, cheap, selective and stable catalysts, which are constantly being searched for by researchers. Most of the products that can be obtained through the catalytic electrochemical reduction of CO_2_ in water in the presence of catalysts can be seen in Figure 1.

Researchers have studied a fairly large number of metal complexes that are catalysts for the electrochemical reduction of CO_2_ [5,6,7,8]. Several well-known water-soluble complexes can be listed: Mn polypyridyl complex [9], rhenium tricarbonyl complex with hydroxymethyl groups [10], 1,10-phenanthroline-copper complex [11], iron tetraphenylporphyrin functionalized with trimethylammonium groups [12], nickel cyclam complex [13], iridium pincer complex [14]. However, most catalytically active complexes dissolve only in organic solvents. It leads to the need to add proton donors at a low concentration (3–5% usually). In turn, water itself is a source of protons, moreover, it is the cheapest and most readily available solvent.

Thus, it is important to look for complexes with catalytically active metal centers, but soluble in water. Sodium pectate has the ability to coordinate various metal centers [15,16,17,18,19,20]. Electrochemical and electrocatalytic properties of sodium pectate complexes are very interesting but have not been studied absolutely. Ligands in such complexes are obtained from pectin, a cheap and readily available natural polysaccharide. In addition, polysaccharides themselves exhibit a catalytic effect in the hydrogen evolution reaction in some cases, as has recently been found out [21,22,23,24]. This article is devoted to the sodium pectate complex with copper and its catalytic activity in the CO_2_RR. We discovered that this water-soluble catalyst (PG-NaCu) is highly active in the CO_2_RR and selectively converts CO_2_ to CH_4_ as product in water solutions.

It is well known that copper electrodes, as well as nanostructured copper and copper oxides, promote the electrocatalytic conversion of CO_2_ to deep reduced products such as methane, ethylene, and ethanol [1,25,26,27]. However, there are a number of examples of the electrocatalytic conversion of CO_2_ to CH_4_, C_2_H_4_, C_2_O_4_ on single copper ions [28,29,30,31,32]. The most common mechanism of such deep reduction of CO_2_ can be noted. In all cases, Cu(I) is the catalytically active site. CO_2_ molecule binds to copper and accepts an electron and a proton with the forming of COOH (Cu(I) becomes Cu(II)). Next, sequential transfer of seven electrons and protons leads to the formation of methane and two water molecules (through the CO and CHO intermediates) [29]. Cu(II) is then reduced back to Cu(I). An interesting effect was observed in one of the works [28], when the distance between Cu centers has a significant impact on a final product of CO_2_RR. If copper ions are distant from each other, CH_4_ is formed. When a pair of copper centers are located close, each of them binds one CO_2_ molecule with the further formation of C_2_H_4_. The formation of C_2_O_4_ in the presence of a dinuclear Cu(I) complex occurs by a similar principle in another article [30].

## 2. Results and Discussion

### 2.1. Synthesis of the Sodium Pectate Complex with Copper

Synthesis of the complex was carried out according to the already known method refs. [15,19,20]. Pectin was dissolved in 1.5 L of water (55 °C), then 0.1 N NaOH solution was added to the pectin, increasing the pH to 9, and then the solution was left for 2 h at 55 °C. Then, a solution of CuSO_4_ with 0.016 mol/L concentration was added to the sodium pectate (PG-Na) solution. In 20–30 min, the target product was precipitated with double volume of ethanol, centrifuged and dried.

**Figure 2:**
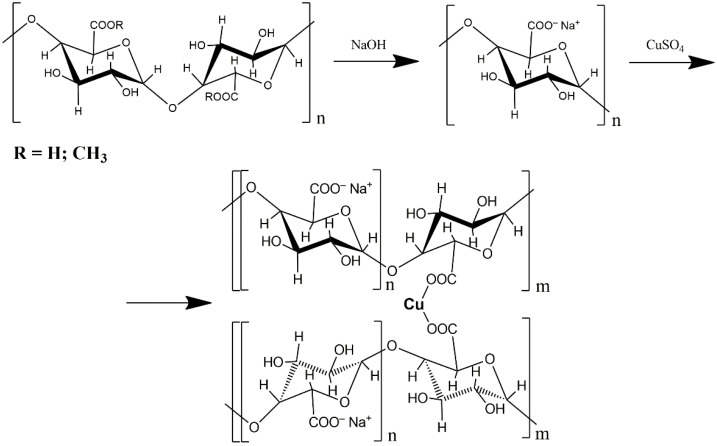
Synthesis of the sodium pectate complex with copper.

For research, we obtained the PG-NaCu complex by using a 20% replacement of sodium ions in sodium pectate with copper ions. The replacement rate was selected in such a way as to both maximize the number of Cu centers and ensure water solubility of the complex. The obtained compound PG-NaCu is amorphous powder. Synthesis and the simplified structure of the complex of sodium pectate with copper is shown in Figure 2.

### 2.2. Electrocatalytic CO_2_RR Tests Using the PG-NaCu Catalyst

We used an electrolysis cell with a large volume of the above-solution space so that it was possible to take samples of the gases formed and carry out their qualitative and quantitative analysis. The area of the working electrode was 1 cm^2^. Sodium phosphate buffer Na_2_HPO_4_/NaH_2_PO_4_ was used as a supporting electrolyte. The electrolysis was carried out at a potential of −1.5 V for 12.5 h in homogeneous conditions and CO_2_ saturated water (the first hour of electrolysis with water saturation with argon).

**Figure 3:**
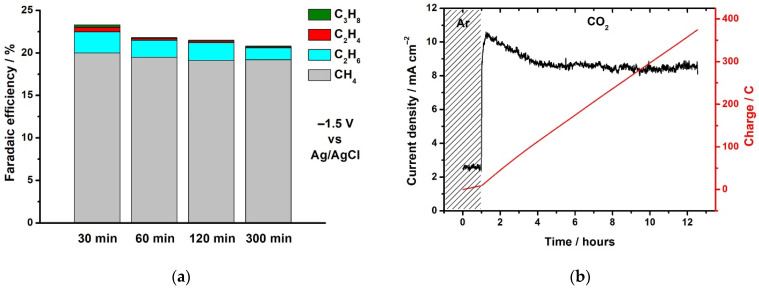
(**a**) Products of the CO_2_RR in the presence of the PG-NaCu complex at −1.5 V vs. Ag/AgCl after different electrolysis times, (**b**) current density (black trace, left) and charge (red trace, right) during bulk electrolysis (E = −1.5 V vs. Ag/AgCl) in homogeneous conditions with the PG-NaCu complex in Ar (the first hour) and CO_2_ saturated water.

Figure 3a shows the result of determining the catalytic CO_2_RR products in the presence of the PG-NaCu complex after different electrolysis times. The calculations of Faraday efficiency were carried out using the values of the concentration of the products found in the selected gas samples and the amount of charge passed through the cell.

(2) $$FE\;=\;\;\frac{Q_{t}}{Q_{f}}\;\times \;100\%$$

(3) $$Q_{f}\;=\;\int_{t_{0}}^{t_{1}}Idt$$

(4) $$Q_{t}=neN_{A}\nu =nF\nu$$

where *n* is the number of electrons in an electrochemical reaction. For example:

(5) $${\mathrm{CO}}_{2}+2e^{-}+2H^{+}\;\to \;\mathrm{CO}\;+{\;H}_{2}O$$

(6) $${\mathrm{CO}}_{2}+8e^{-}+8H^{+}\;\to {\mathrm{CH}}_{4}+2H_{2}O$$

It was found that methane (19.1–20.0%) is the main product of the CO_2_ reduction reaction catalyzed by PG-NaCu. Ethane was also detected (1.2–2.5%). Ethene, propane, and CO are present in insignificant amounts (less than 1% in total). No traces of alcohols (CH_3_OH, C_2_H_5_OH) were found. Hydrogen is also released in large quantities at this potential (76–79%).

The detected products are in agreement with the literature examples of the catalytic CO_2_RR on single copper ions [28,29,30,31,32]. As shown below in Section 2.7 and Section 2.8, Cu(II) in the sodium pectate complex is reduced to Cu(I) acting as a single catalytic site. Apparently, the long distance between Cu centers plays a decisive role in the predominance of C_1_ product over C_2_ and C_3_ products.

Figure 3b shows the chronoamperometry data during electrolysis and the amount of charge passed through the cell. The highest current density after saturation of the solution with carbon dioxide reaches 10.6 mA/cm^2^. Further, within 12.5 h the current density decreases by less than 20% (moreover, the main decrease occurs in the first 3 h of electrolysis in the presence of CO_2_), which characterizes good catalytic stability of the PG-NaCu complex. Such current density is quite high for molecular CO_2_RR electrocatalysts [33], and especially for water-soluble electrocatalysts [9,10,11]. The latter are characterized by current densities of a few mA/cm^2^ or less at close potentials. It should be noted that the immobilization of water-soluble complexes on carbon nanotubes or graphene makes it possible to increase the current density, but it cannot be attributed to the homogeneous catalysis. CO_2_RR catalysts based on metallic copper and copper oxides can exhibit much higher catalytic activity with a current density in the range of 100–400 mA/cm^2^ [25,34]. Having discovered such intriguing properties of the complex, we decided to study it in more detail.

### 2.3. Infrared Spectroscopy

When the Cu pectin metal complex was obtained, the state of carboxyl groups was monitored by infrared (IR) spectroscopy in the range of stretching vibrations of the COO^−^ group (1600–1800 cm^−1^) [35,36,37]. The presence of absorption bands in the IR spectrum of citrus pectin in the range of 1700–1750 cm^−1^, related to the stretching vibrations of carbonyls of carboxyl and ester groups, as well as the presence of characteristic absorption bands in the range of 950–1200 cm^−1^, related to the vibrations of the pyranose ring, confirms the belonging to pectin substances.

In the IR spectrum of sodium pectate (PG-Na) (Figure S1), there is an absorption band in the region of stretching vibrations of the ionic form ν(COO^−^) at 1610 cm^−1^ and there is no absorption band of stretching vibrations ν(C = O) of carboxyl or ester groups at 1745–1750 cm^−1^.

**Figure 4:**
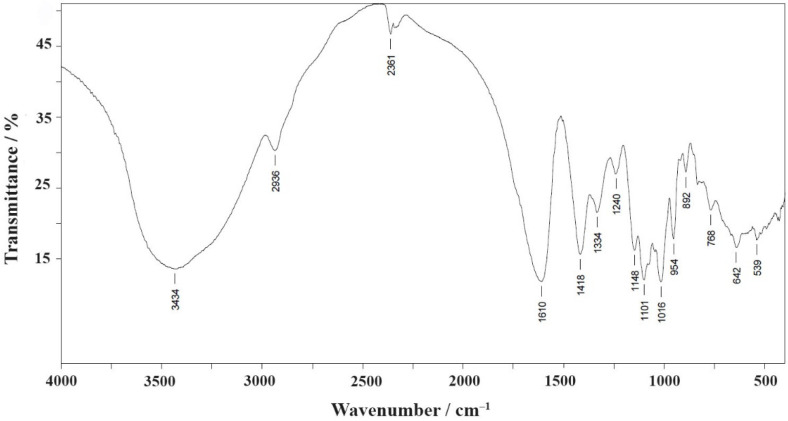
Fourier-transform infrared spectroscopy spectrum of the copper pectin complex (PG-NaCu).

Similarly, the IR spectra of the pectin metal complex PG-NaCu (Figure 4) have characteristic absorption bands of the COO^−^ group. The main characteristic band positions (cm^−1^) for PG-Na and PG-NaCu are shown in Table S1.

### 2.4. Inductively Coupled Plasma Atomic Emission Spectroscopy

The study of the elemental composition of the complex showed that the expected metals content corresponded to the experimental one. The found relative molar content of sodium and copper ions in the PG-NaCu sample was 4.10/1.

### 2.5. Thermal Analysis

**Figure 5:**
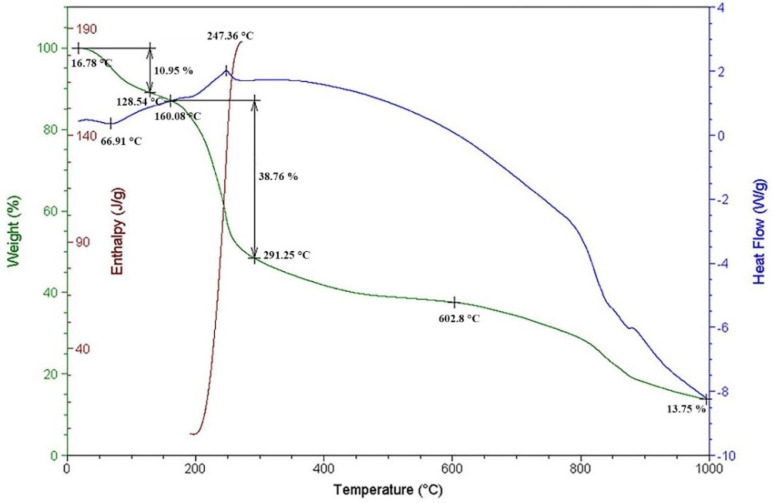
Thermogravimetry and differential scanning calorimetry curves for the sodium pectate complex with copper.

The study of the obtained complex was continued using the combined methods of thermogravimetric analysis (TGA) and differential scanning calorimetry (DSC). As the temperature rises, in the DSC curve for PG-NaCu shown in Figure 5, an endothermic peak can be observed at T ≈ 67 °C. In this case, the weight loss of the sample on the TGA curve was almost 11%. With a further increase in temperature, a peak of the exothermic process is already observed. The temperature of this peak is ≈247 °C, and the corresponding weight loss is already as much as 39%. The enthalpy of reaction, found by integrating the exothermic peak on the DSC curve, was 184 J/g. By analogy with the data obtained for some sodium polygalacturonates and their metal complexes [38,39,40], it can be concluded that the first endothermic peak corresponds to the loss of water by the PG-NaCu sample, and the second exothermic peak is associated with the decarboxylation of the sample and the release of carbon dioxide. All process parameters for PG-Na can be seen in Figure S2. The fundamental difference of PG-Na from the complex is that the sample almost completely loses its mass at a temperature of 1000 °C, while in the case of the copper compound at the same temperature, 14% of the mass remains.

### 2.6. Electron Spin Resonance Spectroscopy

**Figure 6:**
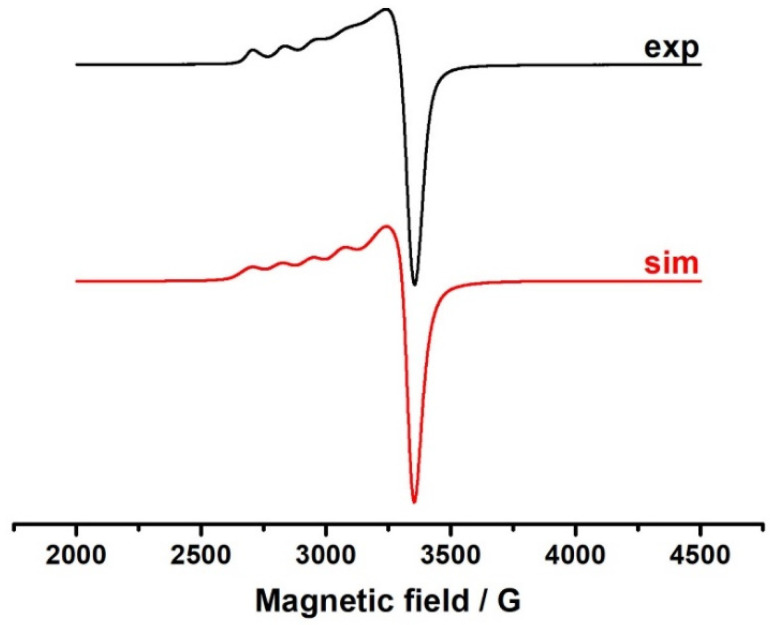
ESR spectrum of the PG-NaCu powder at a temperature of 150 K and its simulation.

The electron spin resonance (ESR) spectrum of the PG-NaCu powder at a temperature of 150 K was obtained and subsequently simulated (Figure 6) to understand the coordination environment of the paramagnetic low-spin Cu (II) 3d^9^ ions in the PG-NaCu complex.

The parameters obtained from the simulation are shown below:

g_1_ = 2.395; a_Cu_ = 122 G; ∆H = 80 G
g_2_ = 2.096; ∆H = 130 G
g_3_ = 2.073; ∆H = 40 G.

The g-factors for the complex are close in their values to the g-factors of the known coordination complexes of Cu(II) with organic acids, in particular, copper citrate [15,41], for which the first coordination sphere of the metal has the structure of a tetragonally distorted octahedron.

### 2.7. Electrochemistry in Homogeneous Conditions (PG-NaCu in Water Solution)

We attempted to carry out both homogeneous and heterogeneous electrocatalysis with the complex. As mentioned in the introduction, the potential of the CO_2_RR in an aqueous medium is much less negative than in organic solvents. However, many molecular catalysts for the CO_2_ reduction reaction are water insoluble. Therefore, researchers often use mixtures of organic solvents with water to carry out homogeneous catalysis [42]. From this point of view, sodium pectate complexes are very convenient, since they are water soluble.

**Figure 7:**
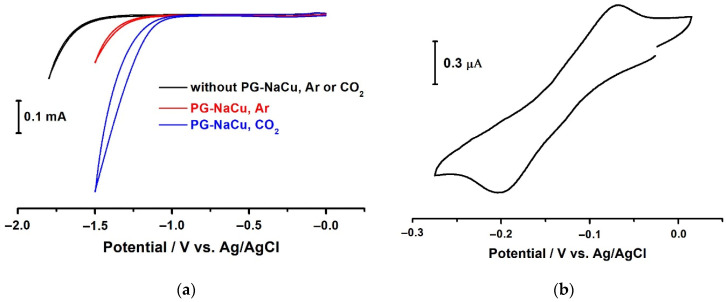
(**a**) CVs recorded at a glassy carbon electrode (GCE) in the presence of the PG-NaCu complex when bubbling argon (red curve) or carbon dioxide (blue curve) through the H_2_O solution, 0.1 V/s. Black line is background in the absence of PG-NaCu, (**b**) CV recorded at a glassy carbon electrode (GCE) in the presence of the PG-NaCu complex when bubbling argon through the H_2_O solution, 0.1 V/s.

First of all, we will focus on the results with the PG-NaCu complex in homogeneous conditions. The E_onset_ potential of hydrogen evolution from water at glassy carbon electrode (GCE) in the absence of the complex is −1.55 V vs. Ag/AgCl both in a solution saturated with argon and in a solution saturated with carbon dioxide (black curve in Figure 7a). The E_onset_ of the same reaction in the presence of the copper complex is already −1.25 V vs. Ag/AgCl (red curve in Figure 7a), i.e., the decrease in overvoltage of this reaction takes place. When the solution is bubbled with carbon dioxide, an even greater shift of the potential E_onset_ occurs, but in this case, one deals with the potential of the multielectron reaction of CO_2_ reduction, which is realized at slightly lower negative potentials than the reaction of hydrogen evolution. The E_onset_ of this reaction reaches −1.05 V vs. Ag/AgCl (blue curve in Figure 7a). It should be noted that the current observed at potentials more negative than the E_onset_ potential in the presence of carbon dioxide are an integral characteristic of two simultaneously occurring and competing processes—the reaction of CO_2_ reduction and hydrogen evolution.

Figure 7a shows the sharp increase in reduction current after E_onset_ in cyclic voltammograms (CVs), but no peaks of the complex reduction are observed. In fact, a quasi-reversible peak is observed at potentials close to 0 V (Figure 7b), but its current is almost a thousand times less than the current at −1.5 V. The low value of the peak current is explained by the slow diffusion of the PG-NaCu molecules to the working electrode due to their large size and high molecular weight. Such a system can be called pseudo-homogeneous, and it combines the advantages of homogeneous and heterogeneous systems. On the one hand, these complexes are quite stable and have many relatively closely spaced copper centers within one molecule, on the other hand, they provide catalytic activity and selectivity inherent in molecular catalysts. As can be seen from the CV, a reduction peak potential of the complex PG-NaCu under homogeneous conditions in water is only −0.2 V vs. Ag/AgCl. Reduction peaks with this potential are typical for the Cu(II)/Cu(I) redox pair. For example, a water-soluble 1,10-phenanthroline-Cu complex (it is an electrocatalyst for the CO_2_RR too) has a reduction Cu(II)/Cu(I) peak with a potential of +0.53 V vs. RHE [11]. Using the equation for electrode potentials converting:

E (vs. Ag/AgCl) = E (vs. RHE) − 0.197 − 0.059 (pH)(7)

We can calculate the potential E_phen-Cu_ (vs. Ag/AgCl) = −0.08 V, which is close to the PG-NaCu reduction peak potential. The solution saturation with carbon dioxide does not lead to any shift in the potential of the peak.

The reduction of copper complexes can sometimes lead to the electrodeposition of metallic Cu (0) or copper oxides on electrodes [43]. There are many examples of both molecular copper catalysts for the CO_2_ reduction [44,45,46] and CO_2_ reduction catalysts based on metallic copper or copper oxides [25,26,27]. We assert that in the case of the PG-NaCu complex and at potentials more positive than −1.5 V (vs. Ag/AgCl), there is no deposition of copper or copper oxide on the glassy carbon electrode. There is some evidence for this.

− Firstly, we do not observe any adsorption peaks on the cyclic voltammograms, which would indicate copper electrodeposition on the electrode.
− Secondly, we carried out a study of the electrode surface before electrolysis, after 30 min and after 12.5 h of electrolysis in the presence of PG-NaCu (Figure S4) using scanning electron microscopy. The electrode was gently washed with deionized water after electrolysis to remove electrolyte residues and only then microscopy was performed. In cases before electrolysis and after 30 min of electrolysis, the surface turned out to be identical without any particles or films. In the case of 12.5 h electrolysis, the presence of a very small number of nanoparticles on the electrode was found. Moreover, energy-dispersive X-ray spectroscopy showed no copper on the electrode surface in all the cases (in the case of 12.5 h of electrolysis, the amount of copper may have been below the sensitivity threshold) (Figure S5).
− Thirdly, we observe a similar catalytic activity of the copper complex under heterogeneous conditions (as will be shown below), where the formation of copper or copper oxide particles or films is unlikely.

### 2.8. Electrochemistry in Heterogeneous Conditions (PG-NaCu in Solid Composite of Carbon Paste Electrode)

Next, we investigated the electrochemical properties of the sodium pectate complex with Cu in heterogeneous conditions. A carbon-paste electrode based on an ionic gel (tri(tert-butyl)(dodecyl)phosphonium tetrafluoroborate) was used. The advantages of this electrode are high electrical conductivity and a wide electrochemical window (5.6 V). This is one of the largest electrochemical windows for ionic liquids, while the paste shows sufficient stability over time and reproducibility of recorded electrochemical signals. The electrode makes it possible to determine the current-voltage characteristics of redox-active insoluble and soluble compounds, which was demonstrated for an insoluble compound poly-tris(μ^2^-1,1′-ferrocenediyl-phenylhydrophosphinato-phenylphosphinato)-iron(III) [47,48]. A glassy carbon electrode with a composite deposited on it mixed with a complex was placed in an electrochemical cell, where water was used as solvent.

**Figure 8:**
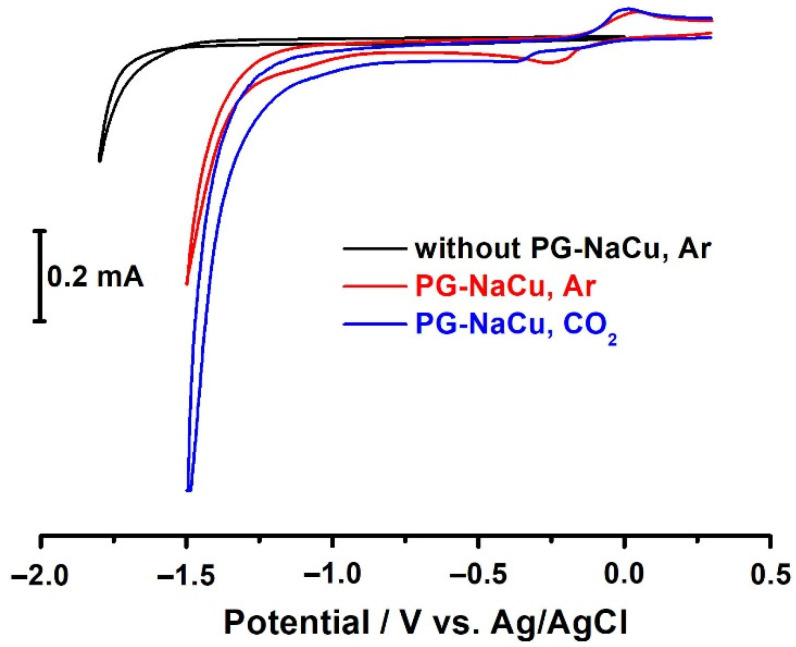
CVs recorded at a carbon-paste electrode (CPE) with the PG-NaCu complex when bubbling argon (red curve) or carbon dioxide (blue curve) through the H_2_O solution, 0.1 V/s. Black line is background at pure CPE.

In the CV diagram, during the reduction in water, a quasi-reversible peak is observed corresponding to the transition of Cu(II) to Cu(I) with a peak potential of only −0.25 V vs. Ag/AgCl (red curve in Figure 8). In general, we observe a result similar to the homogeneous case with a slight difference in the potentials of the reduction and reoxidation peaks. The peak currents are higher than in homogeneous conditions, which is explained by the large number of the PG-NaCu molecules in the paste.

It should be noted that the potential value of the hydrogen evolution reaction shifts to the positive region when a copper complex is added into the carbon paste. The E_onset_ potential of water reduction without the use of PG-NaCu is −1.60 V, the Cu complex shifts the E_onset_ potential by 350 mV (−1.25 V). It is worth noting here that in homogeneous conditions, we obtained a close value for the E_onset_.

When an aqueous solution is saturated with carbon dioxide, the E_onset_ becomes equal to −1.15 V (blue curve in Figure 8) and corresponds to the initial potential of carbon dioxide reduction, and at a potential of −1.50 V, the current density exceeds 14 mA/cm^2^, while the amount of PG-NaCu in the electrode is only 0.1 μg of the substance. There is also a slight shift in the Cu(II)/Cu(I) reduction peak towards negative potentials.

## 3. Materials and Methods

### 3.1. Synthesis of the Sodium Pectate Complex with Copper

We used citrus pectin of the “Classic C-401” brand produced by Herbstreith and Fox (Turnstraße 37, Neuenbürg/Württ, D-75305, Germany) as an organic matrix for copper ions introduction. The measured molecular weight of the citrus pectin is 17.6 kDa. CuSO_4_·5H_2_O, NaOH and other reagents with a purity of more than 99.9% were used for the synthesis.

### 3.2. Fourier-Transform Infrared Spectroscopy

IR spectra were recorded on IR-Fourier spectrophotometer IRS-113 (Bruker, 40 Manning Road, Billerica, MA 01821, USA) with 1 cm^−1^ resolution in the range 400–4000 cm^−1^, the substance being pressed with KBr in tablets.

### 3.3. Inductively Coupled Plasma Optical Emission Spectroscopy

In total, 10 mg of the complex powder was placed in 20 mL of a 0.2 molar solution of HNO_3_ to prepare extracts of the complexes. Na and Cu concentrations were identified in the complex extract using simultaneous inductively coupled plasma optical emission spectrometer (ICP-OES) model iCAP 6300 DUO by Thermo Fisher Scientific Company (168 Third Avenue, Waltham, MA 02451, USA) equipped with a CID detector. Together, the radial and axial view configurations enable optimal peak height measurements with suppressed spectral noises [49]. The concentration of Na and Cu ions was determined, respectively, by the spectral lines 588.995 and 324.754 nm. We used Sc as internal standard (10 ppm in the sample), and all the standards were by the Perkin Elmer corporation.

### 3.4. Thermal Analysis

The thermal decomposition of PG-Na and PG-NaCu was studied by simultaneous thermal analysis (thermogravimetry/differential scanning calorimetry, TG/DSC) in which the variation of the sample mass as a function of temperature and the corresponding heats are recorded. We used a combined TGA/DSC/DTA analyzer SDT Q600 (TA Instruments, USA). The samples (about 12 mg) were placed in corundum crucibles and heated to 1000 °C together with an empty crucible as the reference. The TG/DSC measurements were carried out at a heating rate of 5 K/min in a nitrogen flow of 100 mL/min.

### 3.5. Electron Spin Resonance

ESR measurements were carried out on an ELEXSYS E500 (Bruker) ESR spectrometer of the X-range. ESR spectra were simulated using the WINEPR SimFonia software (Bruker) [50,51].

### 3.6. Electrochemistry

Electrochemical measurements were taken on a BASi Epsilon EClipse electrochemical analyzer (2701 Kent Avenue, West Lafayette, IN 47906, USA). A conventional three-electrode system [52,53,54] was used with glassy carbon as the working electrode, the Ag/AgCl (3 M KCl aqueous solution) electrode as the reference electrode, and a Pt wire as the counter electrode. The pH of solutions undergoing electrochemical studies was maintained with 0.1 M sodium phosphate buffer Na_2_HPO_4_/NaH_2_PO_4_ (pH = 7). It was also a supporting electrolyte. The complex concentration in the solution for electrochemistry under homogeneous conditions was 1 mg/L. The gases were supplied to solutions by the bubbling method. The argon and carbon dioxide purity were higher than 99.99%.

### 3.7. Scanning Electron Microscopy

Microscopy measurements were carried out on an EVO LS-10 scanning electron microscope (Carl Zeiss, Carl-Zeiss-Strasse 22, 73447 Oberkochen, Germany) in high vacuum (HV) mode. SE detector and lanthanum hexaboride cathode were used to obtain surface images. Chemical analysis of the glass carbon surfaces was done using Energy-Dispersive X-ray Spectroscopy detector (Oxford instrument, Tubney Woods, OX13 5QX Abingdon, UK).

### 3.8. Gas Chromatography

Detection and quantification of selected gases (CO and hydrocarbons) were performed by a Crystal 2000 M gas chromatograph (Chromatek, 94 Stroiteley Str., 424000 Yoshkar-Ola, Russia), with a 1 mL sample loop. The gas chromatograph was fitted with two columns (5% NaOH on Al_2_O_3_ and CaA zeolites) and two flame ionization detectors (one of which was fitted with a methanizer). Calibration curves were constructed using certified methane/air and CO/air calibration gas mixtures. Nitrogen was used as the carrier gas. The temperature was held at 60 °C.

## 4. Conclusions

Thus, we were the first to propose the use of copper pectin complexes as selective noble-metal-free electrocatalysts for the conversion of CO_2_ to methane (yield of CH_4_ is 20.0%, other C-products are present in minor amounts). In homogeneous conditions this catalyst works on the verge of heterogeneous and homogeneous catalysis. It can be said that it is nanoheterogeneous since it combines the advantages of a molecular catalyst, soluble in water, as well as heterogeneous due to a large molecular weight (it is a natural polymer). The advantages of the catalyst are its stability, selectivity, the ability to achieve good operating current densities 10.5 mA/cm^2^.

## Data Availability

Not applicable.

## Supplementary Materials

The following are available online, Figure S1: Fourier-transform infrared spectroscopy spectrum of sodium pectate (PG-Na), Table S1: The main characteristic band positions (cm^−1^) for the PG-Na and the PG-NaCu (20%), Figure S2: Thermogravimetry and differential scanning calorimetry curves for sodium pectate (PG-Na), Figure S3: Temperature dependence of the electron spin resonance spectrum of the sodium pectate complex with copper, Figure S4: SEM images of the glass carbon electrode surface before electrolysis (**a**), after 30 min (**b**) and after 12.5 h (**c**, **d**) of electrolysis at −1.5 V vs. Ag/AgCl in the presence of the PG-NaCu. The electrode was gently washed with deionized water after electrolysis to remove electrolyte residues and only then microscopy was performed, Figure S5: Energy-dispersive X-ray spectroscopy of the glass carbon electrode surface before electrolysis (**a**) after 30 min (**b**) and after 12.5 h (**c**) of electrolysis at −1.5 V vs. Ag/AgCl in the presence of PG-NaCu. Carbon is detected in all cases. Very weak oxygen peak is detected in the case of 12.5 h of electrolysis.

## Sample Availability

Samples of the compounds are not available from the authors.
