# Supplementary material for: A Water-Soluble Sodium Pectate Complex with Copper as an Electrochemical Catalyst for Carbon Dioxide Reduction
Source: Molecules. 2021 Sep 11;26(18):5524. doi: 10.3390/molecules26185524 (PMC8470637; doi:10.3390/molecules26185524)
Supplement: Supplementary file 1 [file molecules-26-05524-s001.zip › molecules-1346754-supplementary.pdf]

molecules

Supplementary Material

Water-soluble sodium pectate complex with copper as an electrochemical catalyst for the carbon dioxide reduction

Kirill V. Kholin [1,2,*], Mikhail N. Khrizanforov [1], Vasily M. Babaev [1], Guliya R. Nizameeva [3], Salima T. Minzanova [1], Marsil K. Kadirov [1], Yulia H. Budnikova [1,3]

[1] Arbuzov Institute of Organic and Physical Chemistry, FRC Kazan Scientific Center, Russian Academy of Sciences, Kazan 420088, Russia; khrizanforov@gmail.com (M.N.K.); babaev@iopc.ru (V.M.B.); minzanova@iopc.ru (S.T.M.); kamaka59@gmail.com (M.K.K.); yulia@iopc.ru (Y.H.B.)

[2] Kazan National Research Technical University named after A.N. Tupolev - KAI, Kazan 420111, Russia

[3] Kazan National Research Technological University, Kazan 420015, Russia; guliya.riv@gmail.com

* Correspondence: kholin06@mail.ru

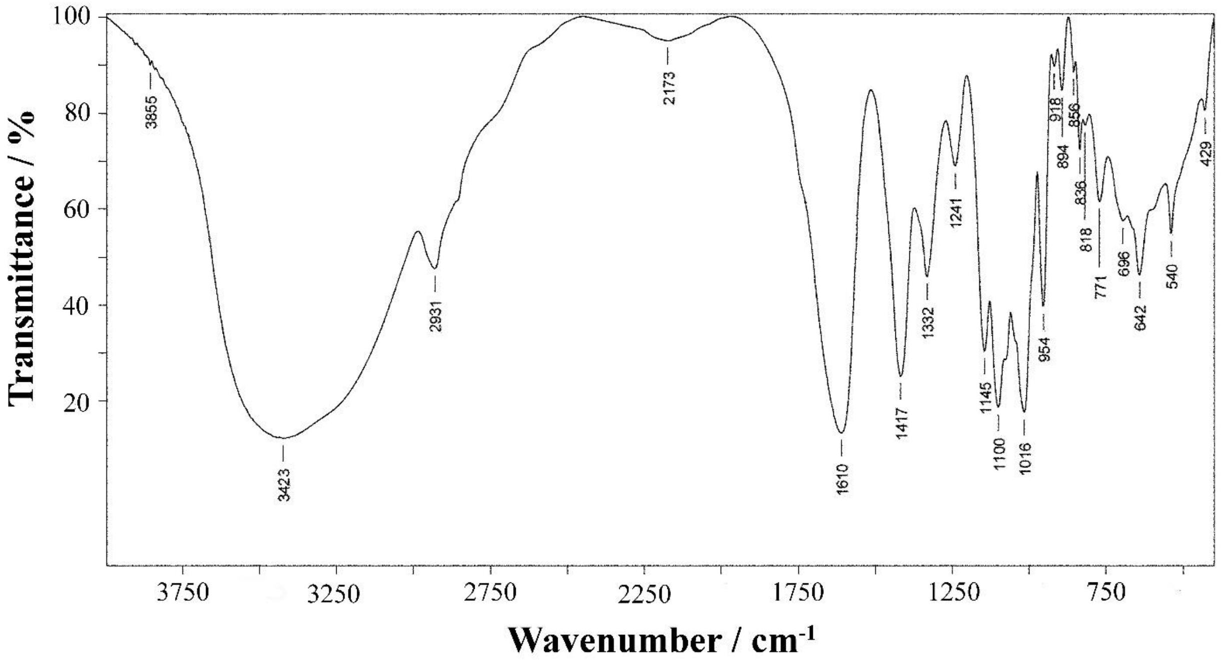

Figure S1. Fourier-transform infrared spectroscopy spectrum of sodium pectate (PG-Na).

Table S1. The main characteristic bands positions (cm^{-1}) for the PG-Na and the PG-NaCu (20%).

Types of vibrations	PG-Na	PG-NaCu
ν (OH), ν (H_2O)	3423	3434
ν (CH), ν (CH)	2931	2936
ν (C=O), ν (C=O)	-	-
ν COO^-, δ (H_2O)	1610	1610
δ_{as} (CH_3)	-	-
δ_s (CH_3)	-	-
ν(C-O), δ (OH),	1417	1418
δ (CH)	1332	1334
δ (OH), δ (CH)	1241	1240
ν (C-O-C)	1145	1148
ν (C-C) (C-O)	1100	1101
ν, δ (C-OH)	-	-
ν (C-C) (C-O)	-	-
ν (C-C) (C-O)	1016	1016
γ (OH)	954	954

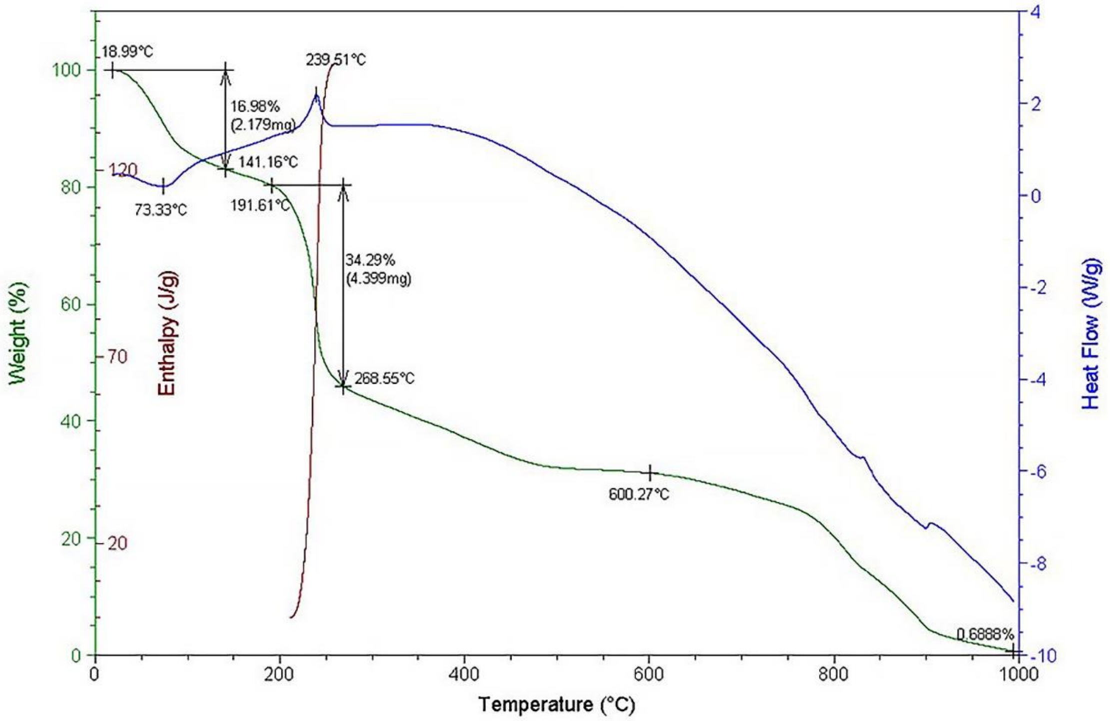

Figure S2. Thermogravimetry and differential scanning calorimetry curves for sodium pectate (PG-Na).

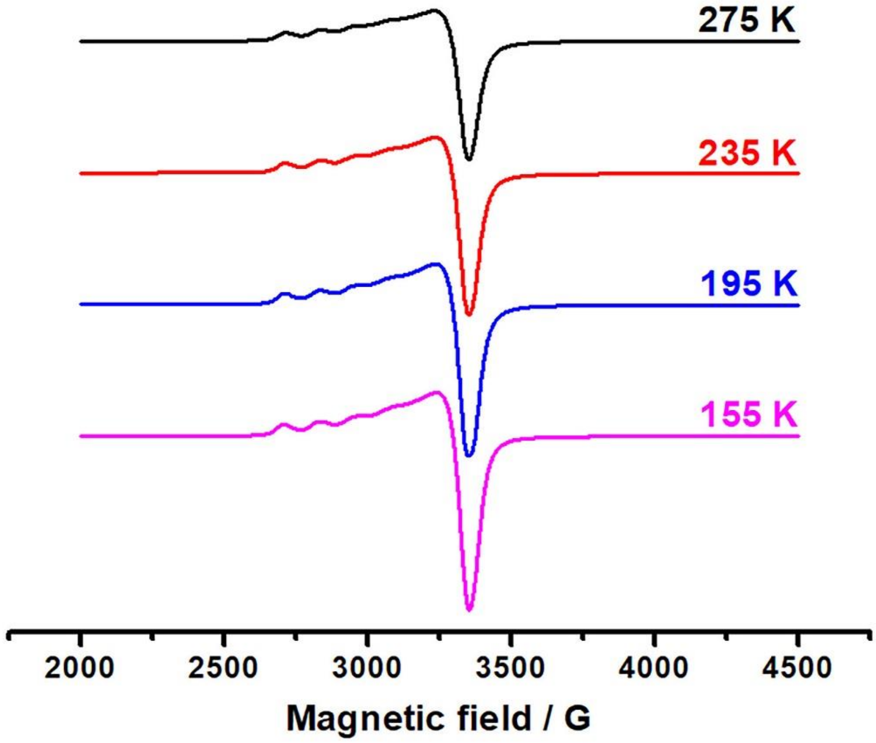

Figure S3. Temperature dependence of the electron spin resonance spectrum of the sodium pectate complex with copper.

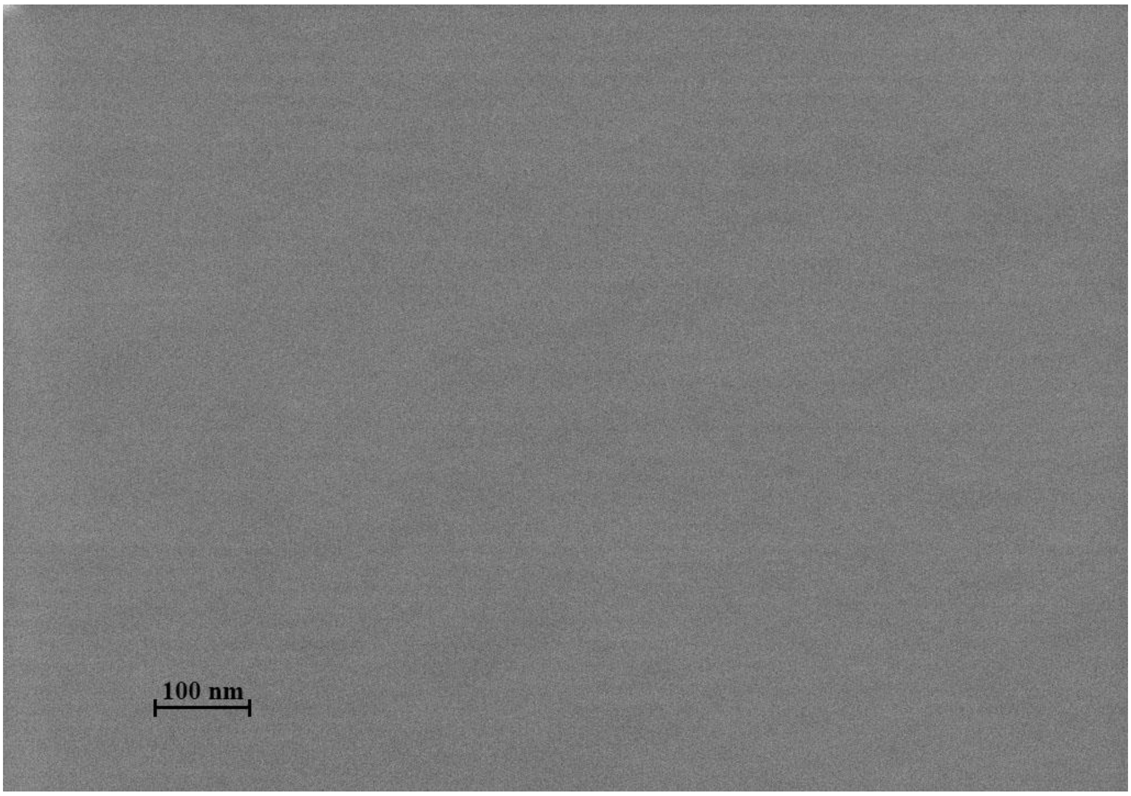

a

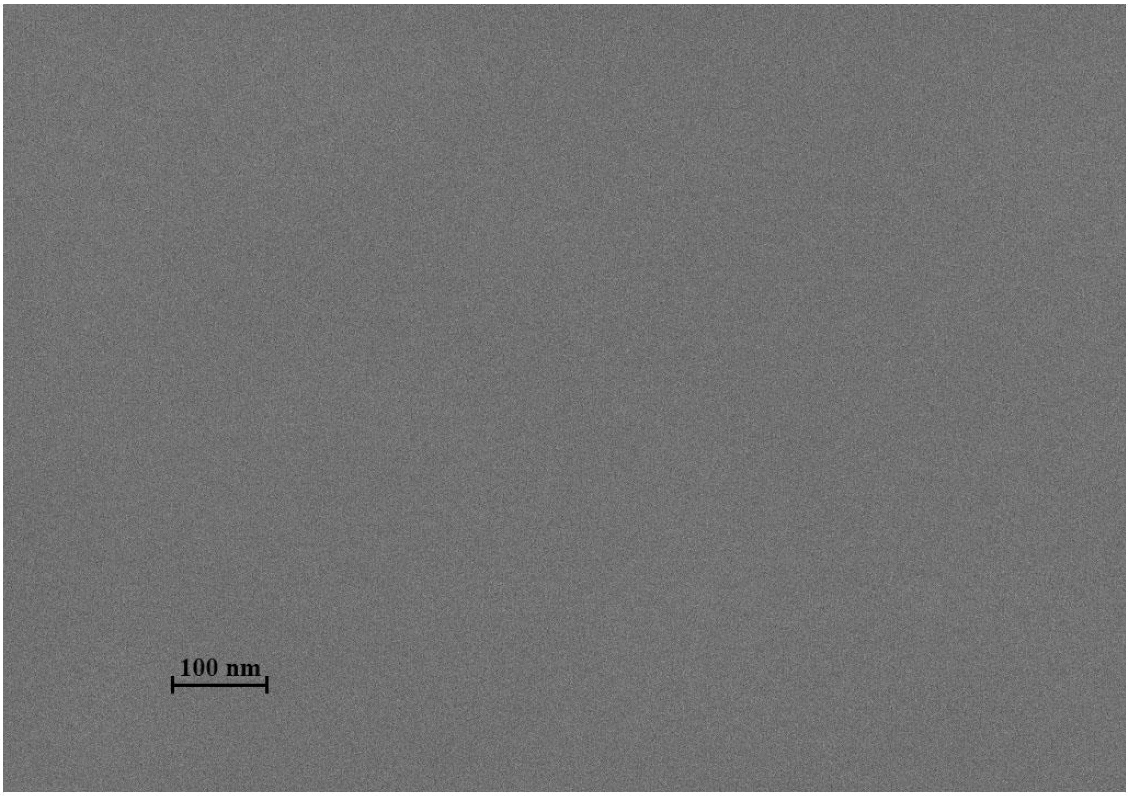

b

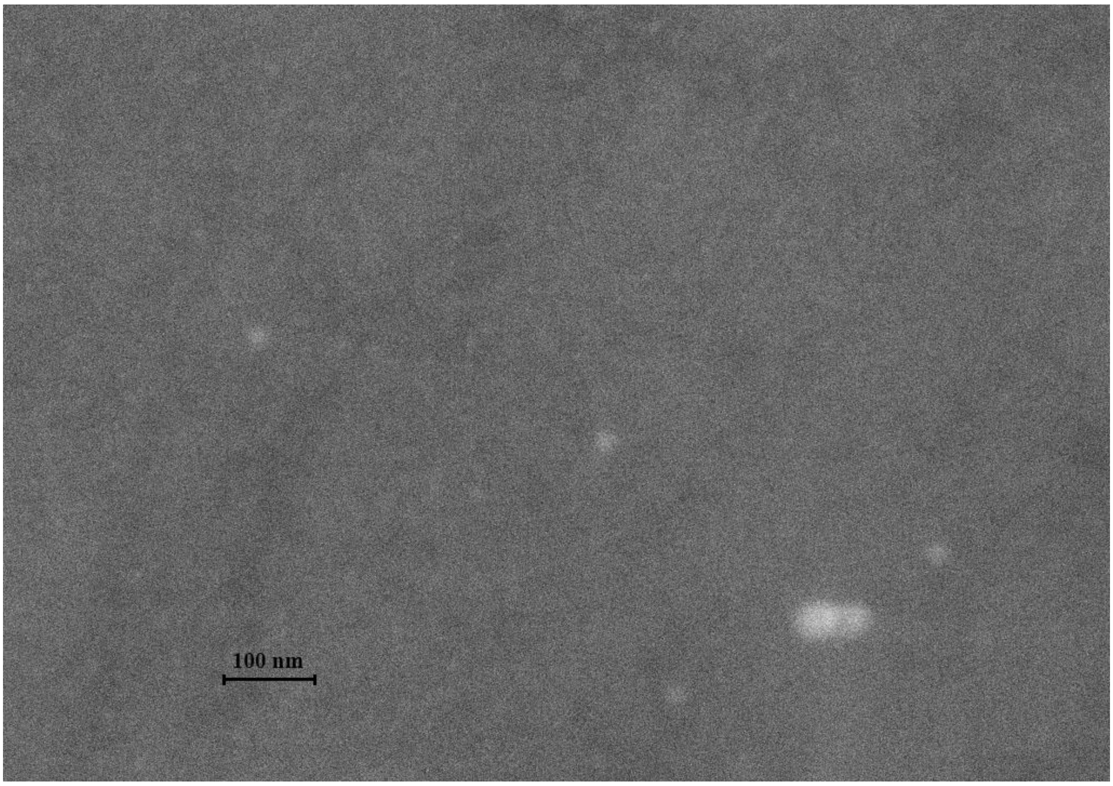

c

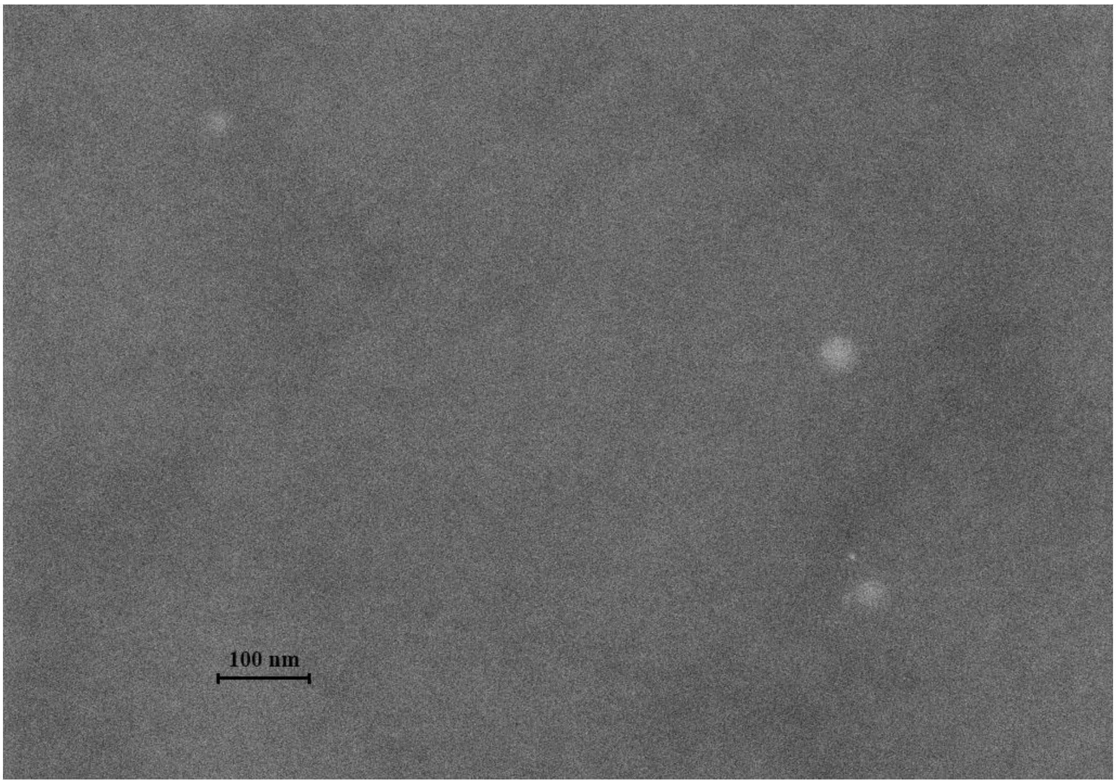

d

Figure S4. SEM images of the glass carbon electrode surface before electrolysis (**a**), after 30 min (**b**) and after 12.5 hours (**c**, **d**) of electrolysis at -1.5 V vs. Ag/AgCl in the presence of the PG-NaCu. The electrode was gently washed with deionized water after electrolysis to remove electrolyte residues and only then microscopy was performed.

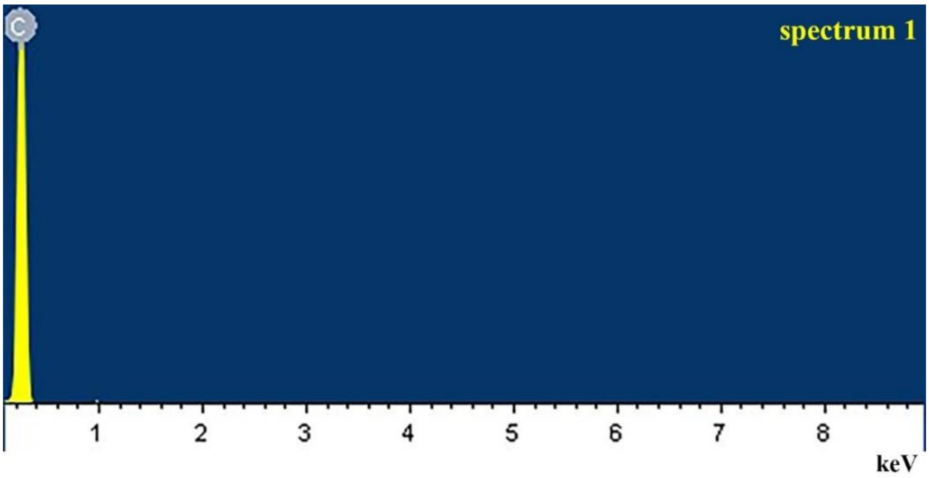

a

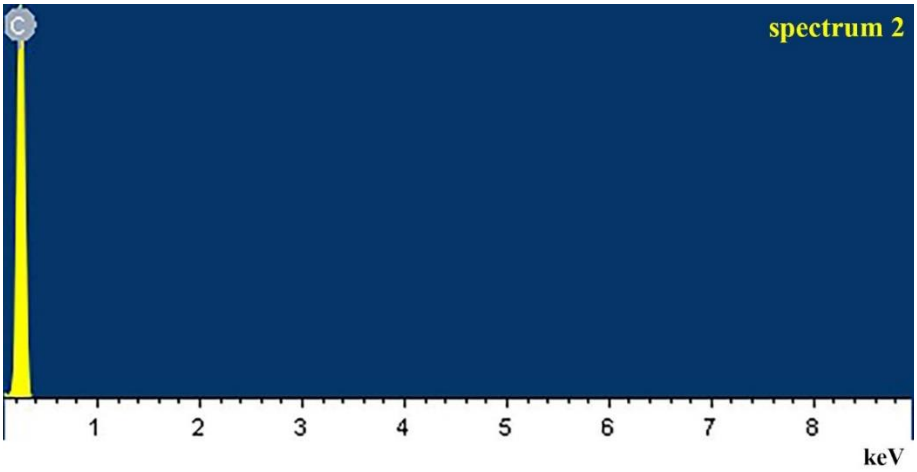

b

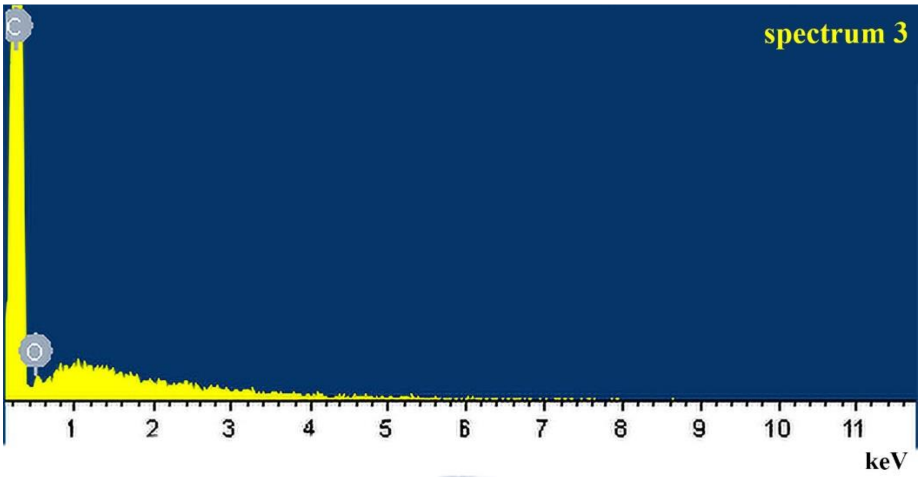

c

Figure S5. Energy-dispersive X-ray spectroscopy of the glass carbon electrode surface before electrolysis (**a**) after 30 min (**b**) and after 12.5 hours (**c**) of electrolysis at -1.5 V vs. Ag/AgCl in the presence of PG-NaCu. Carbon is detected in all cases. Very weak oxygen peak is detected in the case of 12.5 hours of electrolysis.
